# What Parents of Children Born with a Cleft Lip and/or Palate Want to Know About the Care for their Child

**DOI:** 10.1177/10556656241227355

**Published:** 2024-01-18

**Authors:** F.A.C.J. Heijsters, M.D. van Eick, F. van Nassau, M. Bouman, Corstiaan C. Breugem, M.C. de Bruijne, M.G. Mullender, J.P.W. Don Griot

**Affiliations:** 1 Amsterdam UMC location Vrije Universiteit Amsterdam, Department of Plastic, Reconstructive and Hand Surgery, De Boelelaan 1117, Amsterdam, the Netherlands; 2 1229 Amsterdam Public Health Research Institute, Quality of Care, Amsterdam, the Netherlands; 3 Amsterdam UMC location Vrije Universiteit Amsterdam, Department of Public and Occupational Health, De Boelelaan 1117, Amsterdam, the Netherlands; 4 1229 Amsterdam Public Health Research Institute, Amsterdam, the Netherlands; 5 Amsterdam UMC location University of Amsterdam, Department of Plastic, Reconstructive and Hand Surgery, Meibergdreef 9, Amsterdam, the Netherlands; 6 Amsterdam Research Institute Reproduction and Development, Amsterdam, the Netherlands

**Keywords:** patient-centered care, patient satisfaction, experiences, cleft lip and/or palate, qualitative, communication, doctor-patient relationship, digital communication, patient information, multidisciplinary care

## Abstract

**Objective:**

Adequate health information that matches the needs of care recipients is a prerequisite for patient-centered care. To facilitate the provision of tailored and timely information, it isimportant to understand the information needs of parents of children and adolescents with cleft lip and/or palate (CL/P) themselves, and in addition they were asked how they experienced the provided care-related information.

**Design:**

A cross-sectional study employing questionnaires and semi-structured interviews.

**Setting:**

Participants were recruited from a cleft palate-craniofacial care unit in a major tertiary hospital in the Netherlands.

**Participants:**

Participants were parents or guardians of children with CL/P, and two adolescents with CLP. They were recruited through the outpatient clinic during multidisciplinary consultation or after clinical admission.

**Results:**

In total, fifty-five questionnaires were completed by parents or guardians and eleven interviews were conducted with nine parents of children with CL/P and two adolescents with CL/P. In general, participants reported to be satisfied with provided information during hospital admission or multidisciplinary cleft team consultations (mean 8.0, scale 0–10). In addition, 25.5% (n = 14) indicated that information to prepare for hospital admission was lacking (eg, practical information). Thematic qualitative analysis yielded five main information needs: 1) Clear communication during the care process, 2) Overview of the care trajectory, 3) Specific care plan information, 4) Presentation of information and 5) Guidance and support.

**Conclusions:**

Our findings emphasize the importance of gaining insights into wishes and information needs from care recipients who can provide insights in their information needs. With these findings, information provision should be redesigned to improve and to foster the further transition to family-centered care.

## Introduction

Cleft lip and/or palate (CL/P) is the most prevalent congenital craniofacial diagnosis worldwide.^1,2^ Patients often follow an intensive and long-term care path, with the possibility of the care path commencing even before birth until early adulthood and beyond. The diagnosis can be subdivided in (1) cleft lip (CL), (2) cleft palate (CP), and (3) cleft lip and palate (CLP). Since every child born with a cleft is unique, treatment needs to be individualized. Generally, CL/P care is going through a transition towards more family-centered care, which entails more intense involvement of parents, guardians and patients in the care process and decision-making.^3–5^ The family-centered care theory emphasized collaboration and partnership between healthcare providers, patients and their families. Core elements include recognizing and respecting the diverse perspectives of patients and families, fostering open communication, and integrating their values and preferences into the decision-making process for more comprehensive and personalized care.^5–9^

Previous studies have investigated parental needs during the cleft care pathway. According to Nelson et al., parents were positive about their child receiving cleft care when they perceived their healthcare professionals were competent and trustworthy, communicated well, and when they as parents were actively involved in the whole treatment.^10^ Cleft care was perceived less positively when information delivery during the treatment was perceived to be incomplete. Kuttenberger et al. found that parents of children with CL/P requested information about surgery, procedures around feeding their child, and underlying causes of clefts in the initial phase of the cleft care treatment.^11^ Furthermore, parents indicated that more individualized information was desired, which also fits with today's society that has higher information demands.^10^ Besides the need for information by parents, patients also wish to be informed about their treatment in order to participate in the decision-making process.^12^ This need grows when they get older.^4^ Pediatric practice is unique in that developmental maturation over time allows for the inclusion of the patients’ opinion in decision-making in clinical practice.^13^ Although the majority of the patients want to participate in surgical decision-making, they often do not have the appropriate knowledge and understanding of their facial difference and surgical options.^4^ These findings call for a transition from parent-centered to family-centered care. Facilitating this transition requires understanding the information needs of both parents and patients, to tailor the information provision.

Currently, patients and their caregivers can be informed about cleft care in multiple ways, e.g., through their health care provider, information folders, literature, internet, watching videos, etc.^14,15^ Since information is not only provided by their healthcare provider and the internet has become an increasingly important source of information, it is important that online information is understandable and reliable for parents and their children. However, online information on cleft care was found to be too complex for parents in one study, while another study found it to be comprehensible for adults.^16,17^

Little knowledge exists on the wishes of patients and their parents regarding their needs for information, the preferences for the way of information provision and the timing. This can be explored by soliciting parents’ experiences, focusing more clearly on information needs during the care pathway.^18^ Therefore, the aim of this study was to determine what information needs were and considered important by parents of patients born with cleft lip and/or palate with regard to their individual care process.

## Methods

### Study Design

A cross-sectional study was conducted, in which parental questionnaires were combined with semi-structured in-depth interviews with parents and adolescents.

### Context

This study took place in Amsterdam UMC (Amsterdam University Medical Centers), one of the largest tertiary centers in the Netherlands. The cleft team participated in the value-based healthcare program for improving their quality of care in Amsterdam UMC, which included improving patient centeredness of care and the redesign of a renewed shared care pathway of two merging care locations.^19^ Therefore, a needs assessment concerning information provision was required. The findings of this study will be used as input for the larger quality improvement program to ultimately enhance the quality of information provision and to better meet the needs of the targeted population.

Patients and their parents are routinely followed up by the multidisciplinary cleft team at several time points throughout their treatment and care process. The frequency depends on the type of cleft. For insight into personal patient records, the hospital has an integrated electronic patient portal available for patients (from the age of twelve) and parents.^20^ At the time of this study, the cleft team was operating from two locations (AMC and VUmc/ACTA). Each location had their own form of the multidisciplinary consultation day, all specialists in one room (AMC) or in a carousel format with two or three specialists at the time (VUmc/ACTA). Clinical admissions and surgeries took place only at location AMC.

### Participants

**Figure 1:**
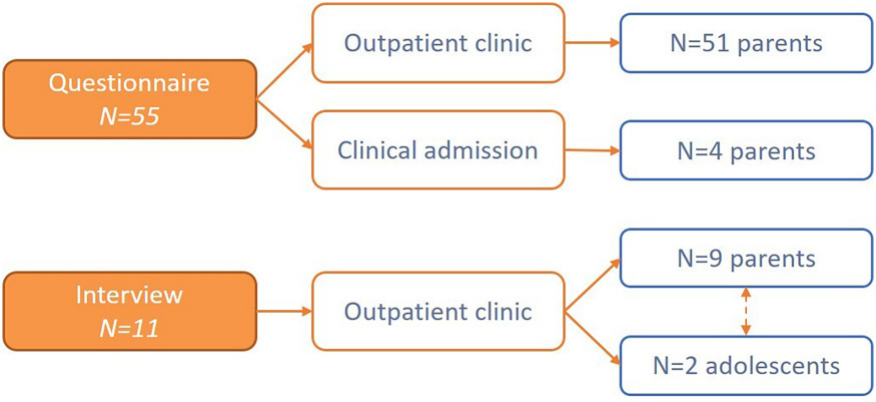
Procedure of participant recruitment.

The study population consisted mainly of parents of children born with different cleft types (CL, CP, CLP). As additional perspective, adolescents (above 16 years) were asked to participate in the interviews to assess their information needs. Participants were included from both locations and varying age stages in the long-term care pathway. Dutch speaking study participants were recruited via the outpatient clinic during multidisciplinary consultation or after clinical admission. Figure 1 shows the procedure of participant recruitment. Data on the response rate were not collected.

### Study Measures

#### Survey

A questionnaire was administered with questions derived from validated questionnaires and additional questions regarding information provision at the hospital. The questionnaire was developed in several discussion rounds by a multidisciplinary team, including researchers specialized in qualitative research methods and clinimetry. Development included an assessment with a patient group in which readability and comprehensability were evaluated. The questionnaire included 28 multiple choice and 3 open-ended questions about: (1) patient characteristics (age, cleft type), (2) experiences of information provision in the current situation (understandability, shared decision-making), (3) preferred situation in information provision (content, sources), (4) improvement suggestions, and (5) digital skills participant (Supplemental file 1). In one of the questions parents were asked to rate the information received: “What rating do you give the information you received before and during this appointment? Select a number from 0–10 (0 = very poor, 10 = excellent)”. In 2020, parents of children with a cleft lip and/or palate, who attended the multidisciplinary consultation at one of the included cleft care locations, were invited to participate in the study by a researcher (FH, LW). Due to the COVID-19 pandemic, recruitment was on hold between April-June 2020. Respondents who showed interest received verbal and written information about the aim and procedure of this study. Questionnaires were filled in digitally on laptops provided by the research team, at the end of a consultation in the outpatient clinic or at home after discharge from the hospital.^21^

#### Interview

Topics for the content of the interview guide were discussed within the multidisciplinary team and pertained to information needs and experiences of care. The qualitative data from the questionnaires were analyzed to be used as input for development of the semi-structured interview guide. In this way data saturation and data comprehensiveness could be ensured.^22^ This resulted in the following themes being addressed: (1) experiences of information provision in the current situation, (2) preferred situation in information provision, (3) improvement suggestions, (4) patient characteristics (age, cleft type) and (5) participant characteristics (age, relationship with patient) (Supplemental file 1). Examples of questions included in the topic guide were ‘What do you find important information?’ and ‘If you were to receive the information again now, what would this ideally look like for you?’ Interview participants were recruited between February and March 2020 during clinical consultation by a specialist nurse. Respondents who showed interest in participation were informed via email about the aim and procedure of the interview. An interview was subsequently scheduled digitally on participants’ preferred date and time between March and April 2020. Interviews were conducted through web-conferencing due to the COVID-19 pandemic.^23^ To include experiences from the patient perspective, participating parents of adolescents (over the age of 16) were asked if their child (born with CL, CP or CLP) wanted to participate individually in our study. If interested, the adolescent was contacted and received written information. When participating, an online interview was scheduled. All audio-recorded interviews were conducted by independent trained researchers (FH, LW) and transcribed verbatim.

### Data Analyses

Given the sample size, only explorative descriptive analyses (ie, means and frequencies) were undertaken on the numeric questionnaire data. Quantitative analyses were conducted in SPSS statistics version 28.0.^24^

Thematic analyses were conducted on the open-text questionnaire answers. Interview data were analyzed using content and thematic analysis in MAXQDA version 2020.^25^ First, three researchers coded six transcripts openly and inductively. During several meetings (FH, FvN) codes were discussed, grouped in overarching codes in a digital tool named Padlet and revised to reach a consensus to ensure quality of the analysis.^26^ The codes used in MAXQDA were described in a final codebook (Supplemental file 2), which was used to code the transcripts by an independent researcher (MvE). After coding the transcripts, categories were formed by deductive axial coding with consensus in coding. These categories were used to form themes and subthemes, described in the results section. In the final step, quotes were selected for illustration. All data were analyzed and presented pseudonymously.

### Ethical Considerations

The study protocol was approved by the Medical Ethical Committee at Amsterdam UMC, Vrije Universiteit Amsterdam (2019.651). The committee considered the study outside the scope of the Medical Research Involving Human Subjects Act (WMO). Participation was voluntary and written informed consent was obtained from each participant before taking part in this study.

## Results

A total of 55 questionnaires were completed by parents. Parents of children with CL, CP or CLP (n = 9 interviews) and two adolescents with CLP (n = 2) were interviewed (Figure 1). The two groups of participants (questionnaires and interviews) overlap. The interviews had a mean duration of 64 min (range 47–78). The patient characteristics are displayed in Table 1. Table 2 presents the demographics of the questionnaire participants.

**Table 1:** Patient characteristics.

	Questionnaires (n = 55)	Interviews*^1,2^* (n = 11)
Cleft type	Cleft lip: 4	Cleft lip: 0
	Cleft palate: 29	Cleft palate: 1
	Cleft lip and palate: 22	Cleft lip and palate: 8
Patients’ age	<1y: 7	<1y: 2
	1–3y: 2	1–3y: 2
	4–6y: 15	4–6y: 0
	7–9y: 9	7–9y: 3
	10–12y: 11	10–12y: 0
	13–15y: 5	13–15y: 0
	16–22y: 6	16–22y: 2*^2^*

*^1^In two of the interviews both parents (ie, father and mother) participated.*

*^2^Both, parent and their child (adolescent), participated in this study.*

**Table 2:** Demographics of 55 questionnaire participants (parents).^27^

	Parents n (%)
Education level^1^	
Low	30 (54.5)
High	25 (45.5)
Native language	
Dutch	49 (89.1)
Non-dutch	6 (10.9)
Digital skilled ^22^	
Yes	46 (83.6)
No or with help	9 (16.4)

*^1^ Low: vocational education or lower; High: university education*

**Figure 2:**
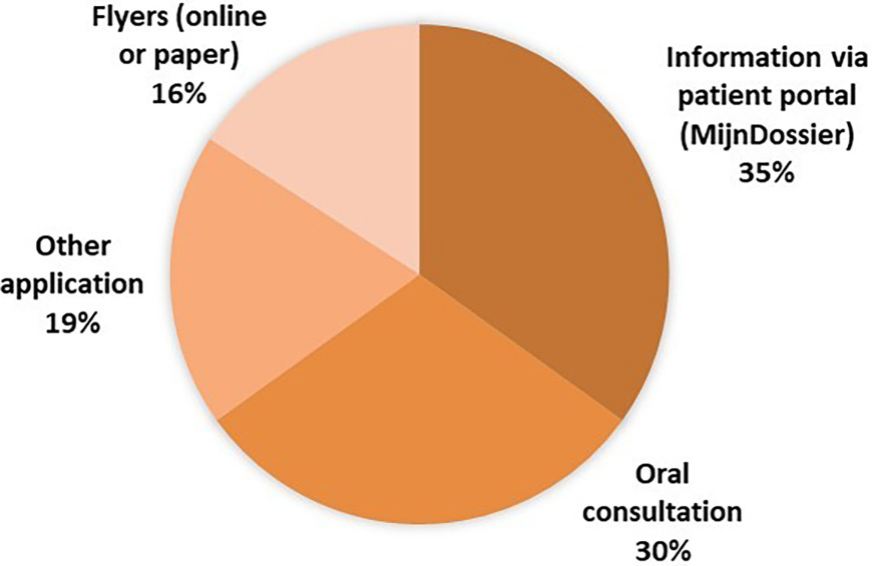
Recommended sources of information cited by parents.

Parents rated the information given during clinical admission (for palate repair or pharyngoplasty) or multidisciplinary consultation on average 8.0 on a 0–10 score, where 0 indicated very poor and 10 indicated excellent (range 2–10). In addition, 25.5% parents (n = 14) indicated that information was lacking in preparation for clinical admission, such as practical information about what to expect, and what to bring to the hospital. Figure 2 showed which other additional information sources were mentioned to be relevant. Overall, 81.8% (n = 45) of the parents reported having a clear view of what to expect during the consultation or admission. In addition, 85.5% (n = 47) indicated that their healthcare professional explained the information in an understandable way during the consultation and 96.4% (n = 53) found hospital folders well understandable. Moreover, 89.1% (n = 49) of the parents felt that they participated in the decision-making process, with 15 respondents (27.2%) indicated that shared decision-making was not necessary, and 34 respondents (61.8%) described being able to fully participate in decision-making.

Thematic analysis of the qualitative interviews yielded five main themes (Table 3).

**Table 3:** Themes and subthemes of qualitative analysis regarding information needs.

Themes	Main Themes	Subthemes
THEME 1	Clear communication during the care process	Communication in between appointments
		Aligned communication with parents
		Communication between healthcare professionals
THEME 2	Overview of the care trajectory	Information structure for expectation management
		Overview of total care pathway
		Care trajectory after the child's age of twelve
THEME 3	Specific care plan information	Diagnosis and consequences of CL/P
		Information in preparation for care
		Information towards child
THEME 4	Presentation of information	Usage of current patient portal
		Accessible and accurate information sources
		Reliability and quality of provided information
		Visualization of information
		Digitalization of information
THEME 5	Guidance and support	Point of contact for support
		Psychological support
		Experiences from peers

## THEME 1: Clear Communication During the Care Process

*Communication in between appointments.* Most parents indicated that there was little communication with their healthcare professionals in between appointments, but when they reached out to the multidisciplinary cleft team, they were satisfied with the accessible communication. However, a few parents highlighted a need for more involvement from the cleft team in between appointments. This need was especially common among parents further along the care pathway (ie, having older children) when appointments became less frequently.

Yes, a phone call would be nice. Even just once a year for example. That way, if you a question, even something seemingly silly, you can discuss it briefly. At the moment, if a question doesn’t feel significant enough, then you keep walking around with it. – Parent of sixteen-year-old child with unilateral CLP

*Aligned communication with parents.* During consultations, communication of different healthcare professionals with parents was not always aligned. According to multiple parents, the information received from different healthcare professionals was not uniform which caused confusion for parents.

You have to massage the lip part,’ then another says, ‘You must do nothing with it,’ yet another says, ‘You have to massage for so many weeks,’ (…) so, there is no clear answer in my opinion. And that (…) can be quite confusing. – Parent of six-month-old child with bilateral CLP

Even though information from different healthcare professionals was not always aligned, the majority of the parents and adolescents were satisfied with the professionals in the cleft team from Amsterdam UMC. For example, one parent mentioned that from the very beginning he felt he was in good hands.

*Communication between healthcare professionals*. The majority of parents did not experience clear communication between different healthcare professionals. Some even had the feeling that they themselves had to pass-through the information from one healthcare professional to the other. Parents also reported that information was not always clearly reported in their child's patient file. Some parents had to repeat their stories multiple times. Other parents mentioned that information registered by one healthcare professional could not be found in the patient file by the other professional.

## THEME 2: Overview of the Care Trajectory

*Information structure for expectation management.* The majority of the participants expressed a need for structured information and communication in their care trajectory. Furthermore, one of the adolescents with CLP would have preferred more involvement in the information provision about the treatment options.

*Overview of total care pathway.* Multiple parents highlighted the absence of a general overview of the care trajectory for children with CL/P. Some other parents indicated that a general path was only outlined with the healthcare professional during one of the first consultations after birth.

Parents indicated that information is generally provided on all types of cleft. However, the majority of parents would prefer personalized information on the specific cleft type of their child for a better understanding of the care trajectory.

Yes, I would specify it for each type. Because it does make a difference what you need to know when the palate is open or closed. – Parent of five-month-old child with unilateral CLP

*Care trajectory after the child's age of twelve*. When children were over twelve years old, some respondents experienced ambiguity regarding appointments in the care trajectory. One of the adolescents with CLP mentioned that not much was explained about surgical options after 12 years old and decisions after turning eighteen years old.

At that point [after 12 years] you also lose the schedule a bit. Now I wonder, ‘When do they want to see him again?’ because I don’t know anymore. I now have nowhere to check when his next visit should be. – Parent of sixteen-year-old child with unilateral CLP

## THEME 3: Specific Care Plan Information

*Diagnosis and consequences of CL/P*. The period between prenatal diagnosis and the first consultation at Amsterdam UMC created uncertainty for many parents. During this period, parents would also have preferred receiving additional information on the particular cleft diagnosis of their child.

Since the care trajectory for patients with CL/P can differ among Dutch academic hospitals, some parents reported a need for clear and visual information about the CL/P care pathway in their hospital specifically.

*Information in preparation for care.* Parents’ preparedness in the care trajectory was expressed during (1) consultation, (2) admission, (3) surgery, and (4) aftercare. During consultation before and right after birth, parents felt well informed and reassured about the care trajectory by the cleft team. Some parents were informed about the proceedings of the multidisciplinary consultation on the spot, but would have preferred to receive the information prior to their visit. This was primarily the case when it was the first consultation at Amsterdam UMC.

The majority of parents mentioned that practical information was absent in various stages of the care pathway. Some parents mentioned they were not informed on any practical tools, such as the Habermann-teat, or did not receive any tips for breastfeeding. Parents indicated a need for more practical information on what to bring to the hospital and what to expect in that specific stage of the care trajectory.

No, we were given no [practical] preparation for the operation. Actually we just got the call that we had to visit the hospital. I then called the nurse myself because I had no idea how such an admission works. – Parent of five-month-old child with unilateral CLP

During hospital admission, opinions about preparedness differed among the parents. Some parents indicated to be well informed and know what to expect during the admission period. Other parents were less prepared for what to expect during the surgical procedure and the number of doctors entering the room during hospital admission. The uncertainty seemed to be linked to the first hospital admission specifically. For later admissions, these parents felt more prepared by knowing what to expect.

The majority of parents felt well informed before an operation and felt confident about the surgical procedure. Furthermore, most parents were satisfied with the timing of the information. Parents were less content with ambiguities in information on waiting times and protocols and expressed a need to clarify this.

During the aftercare period, some patients suffered from complications. These parents indicated a need for more information on possible complications from the multidisciplinary cleft team on discharge from the hospital.

*Information towards child*. When disseminating information to children, the content and timing should be taken into account according to most parents. Furthermore, one of the adolescents with CLP expressed a need for more information on psychological challenges and support for both parents and children.

(…) a lot is discussed about the trajectory in terms of treatment but in my opinion there was very little discussion about the psychological part, about bullying, and about the different school ages; how should we deal with that? – Seventeen-year-old adolescent with unilateral CLP

All parents informed their child about CL/P at an early stage of the care process. Furthermore, they indicated that children were involved in the consultation with the healthcare professionals early on in the care process.

The moment it comes down to communicating and explaining something, it would be helpful to not only take the patient seriously, but to also bring the patient to the center of the conversation. – Parent of seven-year-old child with bilateral CLP

According to the majority of the parents and adolescents, informing children should already start at elementary school when children become more self-aware. The content of the information explained to children should be adapted to their age. Many parents started conversations about the condition with their child at lower elementary school, but an in-depth explanation of the condition started at the age of 12.

I think if you're 12 or above, you should talk to the children themselves. – Sixteen-year-old adolescent with unilateral CLP

## THEME 4: Presentation of Information

*Usage of current patient portal.* The current patient portal (MijnDossier) was used variably among the participants. Some participants indicated that they found the appointment overview convenient and more often accurate than the appointment letter they received. These participants mentioned to use the application of MijnDossier primarily around appointments.

*Accessible and accurate information sources*. A need for accurate information was indicated by the majority of the parents since some parents received outdated information. One parent explained that they thought the obsolete information was probably due to the merge of the hospitals.

Besides the accuracy of the information, the accessibility of information could be improved as well. Some parents indicated that information was sometimes difficult to find online. Another parent highlighted that the use of medical terminologyis not helpful for a patient's understanding of the information.

Multiple parents mentioned that, besides information for patients with CL/P and their parents, information should be available for sharing in their own network, such as with family and friends. Furthermore, one of the adolescents with CLP emphasized the importance of explaining CL/P among peers.

(…) at a certain point everyone starts asking all kinds of questions in your network; friends, family and others. And on the one hand you want to talk about it a lot, because then you can tell your story, but sometimes you are so fed up with it (…). In those moments it would be nice to have something to give the person to read and that they can refer back to on their own. – Parent of one-year-old child with bilateral CLP

All parents indicated the need for clear information sources. The majority of parents used internet as primary source. These parents searched for video's, information at the website from both Amsterdam UMC and other hospitals, Facebook community groups, and blogs with experiences from other parents. However, most parents were very careful with these sources to avoid misleading information and pictures. Other sources used by parents were the Dutch patient association, books about clefts, experiences from other parents in the team with a child with CL/P, the healthcare professionals from the cleft team, and the information evenings organized by Amsterdam UMC. Even though the information evenings were highly valued in general, one of the parents suggested to increase interaction between speaker and parents during these evenings.

So, I actually always got educated by the cleft team itself. That was always sufficient. – Parent of nine-year-old child with unilateral CLP

*Reliability and quality of provided information*. Participants expressed their feelings about the quality and reliability of some information sources, such as YouTube. They mentioned among other things, that quality of the information differed per source. In general, information from the cleft team and provided information at Amsterdam UMC was experienced as high quality information. Yet, the information could sometimes be more concrete and complete according to some participants. Furthermore, participants indicated that hospital-specific information was experienced as more reliable.

*Visualization of information*. The provided information could sometimes be overwhelming or difficult to understand. Many parents highlighted, therefore, a need for visualization (eg, pictures, video's) of complex information to gain a better understanding of the disease.

Furthermore, parents mentioned that it would be helpful if all information is provided at one central place and if reference materials would be provided during the consultations, to be able to read it afterwards. The desired quantity of the information differed a lot among parents. Some parents were satisfied with the information provided, whereas other parents would have preferred more information.

It's not like ‘the more information you get, the more confident you feel’. At least it does not work that way for me. My confidence is instead determined by the course of treatment and whether I feel heard. That is actually more how my trust is built. – Parent of sixteen-year-old child with unilateral CLP

*Digitalization of information.* The majority of the participants preferred digitalized information over information on paper to improve information organization and accessibility. However, the adolescents mentioned that some orally provided information should not be replaced with digitalized tools.

I personally like that [digitalization of care] very much. I also prefer not to receive letters by post, but rather by e-mail, because that way you can find everything more easily and you do not have to keep different folders or be afraid of losing things. So, I do like it a lot, digitalization as much as possible, yes. — Parent of one-year-old child with CP

Besides information about the care trajectory, parents suggested to include all sorts of practical information, option for peer contact, and a section with frequent asked questions in a new application. Furthermore, it would be desirable if a function is incorporated for communication with a healthcare professional of the cleft team.

## THEME 5: Guidance and Support

*Point of contact for support*. Parents expressed a need for a regular point of contact within the cleft team for personal questions. Most parents found this in a cleft nurse and were very satisfied with the accessible communication and information provided.

We also stayed in the hospital for a few days, so we could also go to the nurse there with any questions and we had the direct number of the department in the hospital. So, even when we were at home, if there was anything, we could just call right away and get an answer. — Parent of one-year-old child with CP

*Psychological support*. Parents indicated the need for psychological support during the care pathway of their children. Only some parents indicated a need for psychological help during the care process but all parents agreed that the support should at least be offered. The latter was not the case in practice. Additionally, parents expressed that they were not always satisfied when they received psychological help.

Yeah, I think it might be helpful to talk to someone who can kind of guide you on how to explain this to your child later. Not so much for ourselves, but how do we guide our child through this? — Parent of fourteen-month-old child with CP

Besides support for parents, psychological help for children was needed. Both adolescents did not receive any psychological support during the care process even though they would have appreciated some support if it had been offered.

I never received any information, but if it had been offered it might have been nice. – Sixteen-year-old adolescent with unilateral CLP

*Experiences from peers*. The need for contact with other parents going through the CL/P care trajectory differed among participants. Some parents were aware of the presence of contact-groups but did not feel the need to get in touch, while the majority of parents expressed a wish for contact. However, some parents who were interested in getting in touch with other parents were not always able to find this contact.

But I wanted to have contact with other parents who were also going through this but that wasn’t an option. I did not know how to arrange it myself either. – Parent of sixteen-year-old child with unilateral CLP

For children with CL/P, the need for contact with peers was limited. Both adolescents were aware of the existence of peer-groups but did not feel the need to get in touch with other children with CL/P (yet).

## Discussion

To improve quality of care, we aimed to learn from those experiencing this care. The present study investigated the information needs of parents of children with cleft lip and/or palate, as well as needs of two adolescents. The main needs that were identified were: 1) clear communication during the care process, 2) overview of the care trajectory, 3) specific care plan information, 4) presentation of information and 5) guidance and support.

In general, parents were satisfied with the multidisciplinary cleft team and the overall information provision from the cleft care unit in Amsterdam UMC. The results of this needs assessment showed that most of the information given during a consultation was well understandable by parents. Nevertheless, given the focus in our study on needs, respondents provided practical needs and suggestions how progress could be made in the communication and form, timing and content of information provision during multiple stages of the cleft care trajectory.

The importance of clear communication and appropriate interdisciplinary collaboration in the cleft team was mentioned in the literature, also our results showed that clear and aligned communication was essential for parents and child to feel involved and to avoid confusion.^28,29^ The majority of participants would like to receive the information digitally, preferably offered by their own healthcare provider, which would increase reliability. These results were consistent with literature, where, for example, parents have suggested a ‘step by step’ approach of short videos where specific age/diagnosis topics were addressed as their child progressed through different treatments.^14^ Although parents mentioned that they desired videos that are accessible, trustworthy and positive, attention must be given to the comprehensibility of online information. As described by Wasserburg et al., many online resources were too difficult for patients and parents to understand, which hinders functional health literacy.^17^

The results of this study revealed that the experiences and information needs varied between the different interviewed parents, coinciding with other studies.^30–32^ Which could possibly be explained by the differences in the complexity of the diagnosis, personal preferences in communication style or requirement for amount of information. For example, a healthcare professional should be aware of their communication style and individual patient preferences.^31^ However, our study did also demonstrated that a large proportion of participants mentioned mostly the same information needs, which indicated that these needs should be addressed in clinical practice to contribute to parent and patient-centered care.^33^ The literature also showed that it was important to describe social, mental and physical effects of cleft care in addition to diagnostic information.^34^ Yet the need to receive more personalized information was identified in this study as essential. Herein, an overview of the care pathway must also be provided to meet realistic expectations and ensures elimination of uncertainty among parents. This showed the importance of composing information on daily functioning (social, mental, physical) and health information together with the experts by experience. Involving patients and parents gives an important perspective, which is needed to provide high quality information that match their needs.^32^

Matching information to parent and patients’ needs, which was also evident in our results, as well as to their health literacy, has proven to be an important facilitator of shared decision-making.^33,35^ Family-centered care focusses on a family's needs, values, and preferences, rather than on the underlying diagnosis.^33,36,37^ The importance of family-centered care was confirmed by some of our findings, for example, parents valued involving their child in the conversations during consultations and they expressed a need for suitable information for family members. In addition, several previous studies have indicated that pediatric patients also desire to be involved in the decision-making process and that their preferences should be a driving force in this.^4,38^ For example, our study mentioned the importance of the patient being at the center of the care process. This need for autonomy and involvement increases with age.^39^ Meeting this need will require a further transition to patient- and family-centered care.

### Strengths and Limitations

The current study is one of the first in the field of cleft care to use an iterative approach to describe the information needs of parents with children with CL/P. By combining methods (survey and interviews), experiences from a large group of participants were represented and an in-depth understanding of the needs from a smaller group were included. Furthermore, parents with children at different stages in the CL/P care trajectory were covered to gain insights into the information needs during the entire care process. And lastly, these results can be relevant for other long-term complex conditions in which a multidisciplinary team provides care.

This study also has some limitations. To gain insight in the needs, it was important, due to the merge of the cleft palate-craniofacial care unit, that patients from both locations were included. Experiences with the cleft care may, therefore, not always be comparable for all patients. Secondly, parents were asked about experiences of the entire cleft care trajectory. Since patients from all ages are represented in this study, the prenatal period and first consultations with the cleft team might have been a long time ago for parents with older children. This may have led to recall bias. Thirdly, only two adolescents with CLP were included in the current research. Additional research focusing on children and adolescents is, therefore, necessary to investigate the experiences with cleft care and information needs from a patients’ perspectives. Lastly, the general experiences were scored as positive, but the qualitative results that were described sometimes look negative due to the specific focus and demand on needs.

### Clinical Implications

This study has provided several suggestions for improving clinical practice. First, the need to continue with a central point of contact, the specialist cleft nurse, was found essential by all participants. In addition, it was suggested important to create more awareness of psychological support by healthcare professionals during the care pathway for patients and their parents as its potential problems and effects on general wellbeing were considered to be important. This may require further training and information regarding how to deal with psychosocial issues by healthcare professionals.^30^ In addition, it is important that healthcare professionals ask each individual what they expect and what they, as a parent and patient, need. There were also suggestions to implement while improving the provision of information: more use of graphics, being able to read back information and providing more comprehensive information about the purpose of appointments. All participants responded positively to the use of an informational application during the care trajectory. They indicated that an application could improve insights into the overview of the care pathway. In general, these results described as information needs, recommended resources and clinical implications will also be useful in other cleft palate-craniofacial care units.

### Future Research

Future research should focus on the experiences and information needs of patients from varying ages themselves. To determine what content and form of information provision they would like to have and their desired level of participation in their care pathway, including shared decision-making.

## Conclusion

The present study aimed to gain insight in the needs for information provision for parents of children affected by a cleft lip and/or palate. Although cleft care in general was perceived as “*good*”, it was noticeable that each participant experienced the care process (so far) in their own way, which also translated into different wishes and needs. Active involvement during the care process, in which the child and parents are well-informed based on their personalized care pathway, was expressed as important by participants. This may ensure realistic care expectations. Additionally, this study has provided a number of suggestions for improving the care process and information provision that should be considered in clinical practice.

According to the results of this study, transition to family-centered cleft care needs to be encouraged. So that parents and patients both understand what they can expect and receive proper guidance and support. In this way, the patient can be engaged even more in the care process and during shared decision-making in the future.

## Abbreviations

Amsterdam UMC = Amsterdam University Medical Centers

CL = cleft lip

CP = cleft palate

CLP = cleft lip and palate

CL/P = cleft lip and/or palate

## Supplemental Material

sj-docx-1-cpc-10.1177_10556656241227355 - Supplemental material for What Parents of Children Born with a Cleft Lip and/or Palate Want to Know About the Care for their Child Supplemental material, sj-docx-1-cpc-10.1177_10556656241227355 for What Parents of Children Born with a Cleft Lip and/or Palate Want to Know About the Care for their Child by F.A.C.J. Heijsters, M.D. van Eick, F. van Nassau, M. Bouman, Corstiaan C. Breugem, M.C. de Bruijne, M.G. Mullender and J.P.W. Don Griot in The Cleft Palate Craniofacial Journal

sj-docx-2-cpc-10.1177_10556656241227355 - Supplemental material for What Parents of Children Born with a Cleft Lip and/or Palate Want to Know About the Care for their Child Supplemental material, sj-docx-2-cpc-10.1177_10556656241227355 for What Parents of Children Born with a Cleft Lip and/or Palate Want to Know About the Care for their Child by F.A.C.J. Heijsters, M.D. van Eick, F. van Nassau, M. Bouman, Corstiaan C. Breugem, M.C. de Bruijne, M.G. Mullender and J.P.W. Don Griot in The Cleft Palate Craniofacial Journal

sj-docx-3-cpc-10.1177_10556656241227355 - Supplemental material for What Parents of Children Born with a Cleft Lip and/or Palate Want to Know About the Care for their Child Supplemental material, sj-docx-3-cpc-10.1177_10556656241227355 for What Parents of Children Born with a Cleft Lip and/or Palate Want to Know About the Care for their Child by F.A.C.J. Heijsters, M.D. van Eick, F. van Nassau, M. Bouman, Corstiaan C. Breugem, M.C. de Bruijne, M.G. Mullender and J.P.W. Don Griot in The Cleft Palate Craniofacial Journal
