# Supplementary material for: What Parents of Children Born with a Cleft Lip and/or Palate Want to Know About the Care for their Child
Source: Cleft Palate Craniofac J. 2024 Jan 18;62(5):853–62. doi: 10.1177/10556656241227355 (PMC12106931; doi:10.1177/10556656241227355)
Supplement: sj-docx-1-cpc-10.1177_10556656241227355 - Supplemental material for What Parents of Children Born with a Cleft Lip and/or Palate Want to Know About the Care for their Child [file sj-docx-1-cpc-10.1177_10556656241227355.docx]

| **Themes** | **Topics and Mode of Assessment** | **Reference(s)** |
| --- | --- | --- |
| **Characteristics** | *Survey (multiple choice questions)*  Childs’ cleft type and age at participation (intake questions), mother tongue, health literacy (understanding health information and skills) and educational level. Use of existing digital patient record “MijnDossier” (not heard about it, no, almost never, sometimes, often).  *Semi-structured interview (open-ended questions)*  Childs’ cleft type and age at participation, age and gender of participant, relationship patient-participant. Use of existing digital patient record “MijnDossier” (not heard about it, no, almost never, sometimes, often). | CQi / Picker institute / Chew et al. / NIVEL ^1-4^  and self-constructed questions |
| **Experiences in the current situation** | *Survey (multiple choice questions^*^)*  Assessment of whether a participant was well informed during consultation or clinical admission. Expectations of consultation. Experienced information provision rated from 0 (very bad) -10 (very good). The understandability and usefulness of information provision. The potentially missed information (yes/no, example). Experienced degree of shared decision-making.  *Semi-structured interview*  Introductory: Tell me how you came into contact with the multidisciplinary cleft team.  Assessment of whether a participant was well informed during consultation and how this was experienced (positive and negative experiences). Used sources of information. | CQi / Picker institute / NIVEL / QLQ-INFO26 ^1,2,4,5^  and self-constructed questions |
| **Preferred situation in information provision** | *Survey (multiple choice^*^ and open-ended questions)*  Requirement for additional information. Preferred information provision, divided into content, form and sources.  *Semi-structured interview (open-ended questions)*  Requirement for additional information. Preferred information provision, divided into content, form, sources and timing. | CQi / Picker institute / NIVEL / QLQ-INFO26 ^1,2,4,5^  and self-constructed questions |
| **Improvement suggestions** | *Survey (open-ended questions)*  What improvements in information provision are suggested. They were also given the option to provide other topics and comment as to why.    *Semi-structured interview (open-ended questions)*  What improvements in information provision are suggested.  Demands in digitalization of information provision. They were also given the option to provide other topics and comment as to why. | Self-constructed questions |
| **Digital skills assessment** | *Survey (multiple choice questions)*  A Dutch assessment ‘Quickscan Digital Skills’ containing 6 questions about the digital skills of the participant. Based on this, participants were categorized into one of three groups: 1) yes (using digital tools without support) 2) (using digital tools) with help or 3) no (not using digital tools).  *Semi-structured interview*  Not applicable. Interview was already conducted digitally, which ensured that participant was sufficiently digitally skilled. | Pharos ^6^ |

^*^*Rated on a 1 (totally agree/true) to 4 (totally disagree/untrue) scale. When using another scale, this is specified.*

**References**

1. NFU. Patiëntervaringsmonitor (PEM) https://www.nfu.nl/themas/kwaliteit-van-zorg/patientervaringen-meten

2. AmsterdamUMC. NFU Patiëntervaringsmonitor resultaten 2019

3. Chew LD, Bradley KA, Boyko EJ. Brief questions to identify patients with inadequate health literacy. 2004;

4. NIVEL. Informatiebehoeften van patienten over geneesmiddelen. 2016;

5. Arraras JI, Kuljanic‐Vlasic K, Bjordal K, et al. EORTC QLQ‐INFO26: a questionnaire to assess information given to cancer patients a preliminary analysis in eight countries. *Psycho‐Oncology: Journal of the Psychological, Social and Behavioral Dimensions of Cancer*. 2007;16(3):249-254.

6. Pharos. Quickscan digitale vaardigheden. 2020;
