# Supplementary material for: What Parents of Children Born with a Cleft Lip and/or Palate Want to Know About the Care for their Child
Source: Cleft Palate Craniofac J. 2024 Jan 18;62(5):853–62. doi: 10.1177/10556656241227355 (PMC12106931; doi:10.1177/10556656241227355)
Supplement: sj-docx-2-cpc-10.1177_10556656241227355 - Supplemental material for What Parents of Children Born with a Cleft Lip and/or Palate Want to Know About the Care for their Child [file sj-docx-2-cpc-10.1177_10556656241227355.docx]

**CODEBOEK MIJNEIGENZORGPLAN (SCHISIS)**
*Ervaring en verbetersuggesties informatievoorziening*

Domeinen

[1 Proces 2](#_Toc42196380)

[2 Zorgpad 3](#_Toc42196381)

[3 Inhoud informatievoorziening 5](#_Toc42196382)

[4 Informatiebronnen 7](#_Toc42196383)

[5 Gewenste situatie 8](#_Toc42196384)

[6 Communicatie voor kind 9](#_Toc42196385)

Auteur: Florence Heijsters

Versie: 2.0
Datum: 12 april 2022

# 1 Proces

| Onder het domein proces vallen de volgende onderwerpen:   - De verschillende fases in het zorgtraject schisis die verband hebben met verschillende informatie behoefte (prenataal, geboorte, eerste levensjaar, 1-6 jaar, 6-9 jaar, 9-12 jaar, 12-18 jaar, 18-22 jaar). Denk hiervoor aan 3 belangrijke momenten; ouders beslissen voor kind, kind snapt genoeg om zelf geïnformeerd te worden, kind is oud genoeg om zelf beslissingen te nemen. - Reflectie en evaluatie van het zorgproces: Bespreking van alle positieve en negatieve ervaringen rondom het zorgproces. - Benoemen van suggesties voor verbeteringen van het zorgproces. |
| --- |

| **Domein** | **Subdomein** | **Code in MAXQDA** | **Omschrijving van de code** |
| --- | --- | --- | --- |
| 1 Proces | 1.1 20 weken echo | 1.1 20 weken echo | Alles wat er gezegd wordt over de communicatie en informatievoorziening rondom de 20 weken echo en de nodige vervolgstappen in het zorgproces. |
| 1 Proces | 1.2 Verwijzing | 1.2 Verwijzing | Alles wat er gezegd wordt over verloop van het verwijzingsproces. |
| 1 Proces | 1.3 Prenataal gesprek | 1.3 Prenataal gesprek | Alles wat er gezegd wordt over de ervaringen met het prenatale gesprek en de betrokkenheid van het schisisteam rondom het prenatale gesprek. |
| 1 Proces | 1.4 Rondom geboorte | 1.4 Rondom geboorte | Alles wat er gezegd wordt over het verloop van het proces rondom de geboorte. |
| 1 Proces | 1.5 Eerste afspraak na bevalling | 1.5 Eerste afspraak na bevalling | Alles wat er gezegd wordt over de ervaringen met de eerste afspraak na de bevalling. |
| 1 Proces | 1.6 Operaties | 1.6.1 Voorbereiding | Positieve en negatieve ervaringen van patiënten en ouders die te maken hebben met de voorbereiding op operaties. |
|  |  | 1.6.2 Tijdens opname | Positieve en negatieve ervaringen van patiënten en ouders tijdens een opname in het ziekenhuis. |
|  |  | 1.6.3 Na opname | Positieve en negatieve ervaringen van patiënten en ouders na een opname in het ziekenhuis. |
| 1 Proces | 1.7 Traject na 12 jaar | 1.7 Traject na 12 jaar | Alle ervaringen die genoemd worden over het zorgproces na 12 jaar. |
| 1 Proces | 1.8Aanbieden psychologische hulp | 1.8 Aanbieden psychologische hulp | Alles wat gezegd wordt over het aanbieden of ontvangen van psychologische hulp gedurende het zorgproces. |
| 1 Proces | 1.9 Aanspreekpunt | 1.9.1. Vorm | Alles wat er gezegd wordt over de vorm van aanspreekpunt, zoals email, telefoon, etc. |
|  |  | 1.9.2 Empathie | Alles wat er gezegd wordt over wat de patiënt verwacht qua empathie van de zorgverlener. |
|  |  | 1.9.3 Aanspreekpunt algemeen | Alles wat er gezegd wordt over de behoefte van de patiënt en ouder om 1 aanspraakpunt te hebben. |
|  |  | 1.9.4 Toegankelijkheid | Alles wat er gezegd wordt over de toegankelijkheid/beschikbaarheid van een aanspreekpunt. |
| 1 Proces | 1.10 Afspraak inplannen | 1.10 Afspraak inplannen | Alles wat er gezegd wordt over het proces van een afspraak inplannen. |
| 1 Proces | 1.11 Contact tussen afspraken | 1.11 Contact tussen afspraken | Alles wat er gezegd wordt over contact momenten tussen de afspraken in. |
| 1 Proces | 1.12 Vorm spreekuur | 1.12 Vorm spreekuur | Beschrijving van ervaring en suggesties over de vorm van het spreekuur. |
| 1 Proces | 1.13 Communicatie | 1.13.1 Communicatie VU en derden | Alles wat er gezegd wordt over de communicatie van VU en derden partijen (vb: logopedie) |
|  |  | 1.13.2 Communicatie tussen artsen | Alles wat er gezegd wordt over de communicatie tussen artsen |
|  |  | 1.13.3 Uniformiteit informatie | Alles wat gezegd wordt over de communicatie onderling en daarbij de uniformiteit van informatie. |
| 1 Proces | 1.14 Adoptie | 1.14 Adoptie | Invloed van adoptie op het zorgtraject als operatie in het buitenland is gedaan of nog uitgevoerd moet worden op een latere leeftijd. |
| 1 Proces | 1.15 Patiëntbeleving | 1.15 Algemeen | Algemene opmerkingen over de ervaring of patiëntbeleving van het zorgproces. |
| 1 Proces | 1.15 Patiëntbeleving | 1.15.2 Beslismoment | Alles wat wordt gezegd over de ervaring vanuit patiënten en ouders m.b.t. belangrijke beslismomenten in het zorgproces |
| 1 Proces | 1.16 MijnDossier | 1.16.1. Ervaringen met functionaliteiten | Alles wat wordt gezegd over de ervaring van de functionaliteiten van MijnDossier. |
|  |  | 1.16.2. Toegankelijkheid | Alles wat wordt gezegd over de toegankelijkheid van MijnDossier. |
|  |  | 1.16.3. Gebruiksredenen | Beschrijving redenen en ervaringen met het gebruik MijnDossier. |
|  |  | 1.16.4. Suggesties | Alle suggesties die worden beschreven voor MijnDossier (gebruik en functionaliteiten) |
| 1 Proces | 1.17 Orthodontie | 1.17 Orthodontie | Alles wat wordt gezegd over het proces tijdens en rondom orthodontiebehandelingen |

# 2 Zorgpad/zorgtraject

| Onder het domein zorgpad vallen de volgende onderwerpen:   - Reflectie en evaluatie: Bespreking van alle positieve en negatieve ervaringen rondom het zorgtraject. - Benoemen van suggesties tot verduidelijking van het zorgtraject. - De geïnterviewde krijgt in het interview het gevisualiseerde schisis zorgtraject te zien met een tijdlijn van het gehele traject 0-22jaar. De eerste indruk en ervaringen worden in dit domein meenomen. |
| --- |

| **Domein** | **Subdomein** | **Code in MAXQDA** | **Omschrijving van de code** |
| --- | --- | --- | --- |
| 2 Zorgpad | 2.1 Overzicht | 2.1 Overzicht | Beschrijving van ervaring met het bestaande overzicht van het schisis zorgtraject. |
| 2 Zorgpad | 2.2 Structuur | 2.2 Structuur | Alles wat gezegd wordt over het belang van structuur in het aanbieden van informatie gedurende het zorgtraject. |
| 2 Zorgpad | 2.3 Visualisatie | 2.3 Visualisatie | Alles wat gezegd wordt over het belang van visualisatie in het aanbieden van informatie gedurende het zorgtraject. |
| 2 Zorgpad | 2.4 Personalisatie | 2.4 Personalisatie | Beschrijving in hoeverre de informatievoorziening gedurende het zorgpad is gepersonaliseerd en welke suggesties er zijn. |

# 3 Inhoud informatievoorziening

| Onderstaande factoren vallen onder dit domein:   - Beschrijving van de inhoud van de huidige informatievoorziening. - Benoemen van suggesties tot verbetering van de inhoud van de informatievoorziening - Reflectie en evaluatie: Bespreking van alle positieve en negatieve ervaringen rondom de huidige informatievoorziening |
| --- |

| **Domein** | **Subdomein** | **Code in MAXQDA** | **Omschrijving van de code** |
| --- | --- | --- | --- |
| 3 Inhoud informatievoorziening | 3.1 Schisisteam algemeen | 3.1 Schisisteam algemeen | Alles wat gezegd wordt over de ervaringen met de informatie over het schisisteam (in het algemeen). |
| 3 Inhoud informatievoorziening | 3.2 Informatie verschillende schisisteams | 3.2 Informatie verschillende schisisteams | Alles wat gezegd wordt over het aanbieden van informatie over de verschillende schisisteams in verschillende ziekenhuizen. |
| 3 Inhoud informatievoorziening | 3.3 Type schisis | 3.3 Type schisis | Alles wat gezegd wordt over het aanbieden van informatie over het type schisis. |
| 3 Inhoud informatievoorziening | 3.4 Prenatale fase | 3.4 Prenatale fase | Alles wat er gezegd wordt over informatievoorziening tijdens de prenatale fase. |
| 3 Inhoud informatievoorziening | 3.5 Rondom geboorte | 3.5 Rondom geboorte | Alles wat er gezegd wordt over informatievoorziening rondom de geboorte |
| 3 Inhoud informatievoorziening | 3.6 Operaties | 3.6.1. Timing van informatie | Beschrijving van de ervaring, behoefte en timing van het ontvangen van informatie rondom operaties. |
|  |  | 3.6.2. Operatietechniek | Alles wat er gezegd wordt over de beschrijving of visualisatie van operatietechniek. |
|  |  | 3.6.3. Complicaties | Alles wat er gezegd wordt over het aanbieden van informatie over complicaties tijdens/na een operatie |
|  |  | 3.6.4. Voorbereidend voor opname | Alles wat er gezegd wordt over het aanbieden van informatie ter voorbereiding van een opname |
|  |  | 3.6.5. Tijdens opname | Alles wat er gezegd wordt over het aanbieden van informatie en de ervaringen tijdens opname |
|  |  | 3.6.6. Nazorg | Alles wat er gezegd wordt over het aanbieden van informatie over de nazorg |
| 3 Inhoud informatievoorziening | 3.7 Voeding | 3.7 Voeding | Alles wat er gezegd wordt over het aanbieden van informatie over de voeding na de geboorte. |
| 3 Inhoud informatievoorziening | 3.8 Gehoor | 3.8 Gehoor | Alles wat er gezegd wordt over het aanbieden van informatie over gehoor bij een schisis. |
| 3 Inhoud informatievoorziening | 3.9 Logopedie | 3.9 Logopedie | Alles wat er gezegd wordt over het aanbieden van informatie over de logopedie bij een kind met schisis. |
| 3 Inhoud informatievoorziening | 3.10 Gebit | 3.10.1. Orthodontie | Alles wat er gezegd wordt over het aanbieden van informatie over het gebit en wat de fase met orthodontie bij een kind met een schisis inhoud. |
|  |  | 3.10.2. Kaakoperatie | Alles wat er gezegd wordt over informatie voorziening rondom een kaakoperatie. |
| 3 Inhoud informatievoorziening | 3.11 Psychologie | 3.11 Psychologie | Alles wat er gezegd wordt over de behoefte en informatie voorziening over de psychologische kant bij een kind met een schisis. |
| 3 Inhoud informatievoorziening | 3.12 Verwachtingen m.b.t. vervolgafspraken | 3.12 Verwachtingen m.b.t. vervolgafspraken | Alles wat er gezegd wordt over het aanbieden van informatie wat van invloed is op de verwachtingen met betrekking tot vervolgafspraken. |
| 3 Inhoud informatievoorziening | 3.13 Ervaringen van anderen | 3.13 Ervaringen van anderen | Alles wat gezegd wordt over de informatie die wordt gedeeld over de ervaringen van anderen (lotgenoten). |
| 3 Inhoud informatievoorziening | 3.14 Gesprek met arts | 3.14.1 Ervaring | Alles wat gezegd wordt over de algemene ervaring met het gesprek met arts, inclusief wat er inhoudelijk gezegd wordt. |
|  |  | 3.14.2 Hoeveelheid informatie | Alles wat gezegd wordt over de ervaringen met de hoeveelheid van informatie gedurende het gesprek met zorgverleners. |
|  |  | 3.14.3 Vertrouwen door informatie | Alles wat gezegd wordt over in welke mate de informatie invloed heeft op het vertrouwen. |
| 3 Inhoud informatievoorziening | 3.15 Adoptie | 3.15 Adoptie | Alles wat gezegd wordt over de informatie die wordt aangeboden over het behandelen van een schisis na adoptie. |
| 3 Inhoud informatievoorziening | 3.16 Taalgebruik | 3.16 Taalgebruik | Alles wat gezegd wordt over het taalgebruik in de informatievoorziening. |

# 4 Informatiebronnen

| Onderstaande factoren vallen onder dit domein:   - Beschrijving van de inhoud van de huidige en gewenste informatiebronnen - Benoemen van suggesties tot verbetering van de inhoud, het gebruik en de beschikbaarheid van informatiebronnen - Reflectie en evaluatie: Bespreking van alle positieve en negatieve ervaringen rondom de gebruikte informatiebronnen |
| --- |

| **Domein** | **Subdomein** | **Code in MAXQDA** | **Omschrijving van de code** |
| --- | --- | --- | --- |
| 4 Informatiebronnen | 4.1 Bron algemeen | 4.1.1 Actualiteit | Alles wat er gezegd wordt over kwaliteit en actualiteit van de informatie. |
|  |  | 4.1.2. Toegankelijkheid | Alles wat er gezegd wordt over de toegankelijkheid van de informatiebronnen. |
|  |  | 4.1.3. Verspreiding | Alles wat er gezegd wordt over de mogelijkheid tot verspreiding van kennis en informatie. |
| 4 Informatiebronnen | 4.2 Voorbeelden van bronnen | 4.2.1 Patiëntvereniging | Alles wat er gezegd wordt over ervaringen met bronnen die mensen raadplegen om informatie op te halen. Focus hierbij op de patiëntvereniging. |
|  |  | 4.2.2 Arts | Alles wat er gezegd wordt over ervaringen met bronnen die mensen raadplegen om informatie op te halen. Focus hierbij op de arts en middelen verstrekt vanuit de polikliniek. |
|  |  | 4.2.3 Internet | Alles wat er gezegd wordt over ervaringen met bronnen die mensen raadplegen om informatie op te halen. Focus hierbij op het internet. |
|  |  | 4.2.4 Overig | Alles wat er gezegd wordt over bronnen die mensen raadplegen om informatie op te halen. Overige bronnen worden hierin genoemd. |
|  |  | 4.2.5 MijnDossier | Alles wat er gezegd wordt over de MijnDossier functionaliteit als bron van informatievoorziening |
| 4 Informatiebronnen | 4.3 Voorlichtingsavond | 4.3.1. Aanmelding | Alles wat er gezegd wordt over het aanmeldproces en de vrije keuze rondom deelname aan de voorlichtingsavond. |
|  |  | 4.3.2. Moment | Alles wat er gezegd wordt over de frequentie en timing van de voorlichtingsavond |
|  |  | 4.3.3. Vorm tijdens avond | Alles wat er gezegd wordt over de presentatie vorm tijdens de voorlichtingsavond |
|  |  | 4.3.4. Naslagwerk | Alles wat er gezegd wordt over het beschikbare naslag werk na deelname aan de voorlichtingsavond |
| 4 Informatiebronnen | 4.4 Inhoud bron | 4.4 Inhoud bron | Alles wat er gezegd wordt over de gewenste inhoud van bronnen. |
| 4 Informatiebronnen | 4.5 Vindbaarheid | 4.5 Vindbaarheid | Alles wat er gezegd wordt over de vindbaarheid van informatie. |
| 4 Informatiebronnen | 4.6 Kwaliteit | 4.6 Kwaliteit | Alles wat gezegd wordt over de kwaliteit van de informatie. |
| 4 Informatiebronnen | 4.7 Betrouwbaarheid | 4.7 Betrouwbaarheid | Alles wat gezegd wordt over betrouwbaarheid van de informatie. |
| 4 Informatiebronnen | 4.8 Vorm informatievoorziening | 4.8 Vorm | Alles wat gezegd wordt over verschillende vormen en manieren van het aanbieden van informatie. |
| 4 Informatiebronnen | 4.9 Hoeveelheid informatie | 4.9 Hoeveelheid  (+ oude code  2.5 dosering, kijken waar dit het beste past) | Alles wat gezegd wordt over de hoeveelheid van informatie die wordt aanboden in bronnen. |

# 5 Gewenste situatie

| Onderstaande factoren vallen onder dit domein:   - Beschrijving van de gewenste situatie met betrekking tot de informatievoorziening. - Reflectie en evaluatie: Bespreking van alle positieve en negatieve ervaringen rondom de voorbeelden van de app MediMapp en het zorgpad overzicht. - Beschrijving van inhoud en functionaliteiten die MediMapp in de gewenste situatie moet bevatten. |
| --- |

| **Domein** | **Subdomein** | **Code in MAXQDA** | **Omschrijving van de code** |
| --- | --- | --- | --- |
| 5 Gewenste situatie | 5.1 Digitalisering | 5.1 Digitalisering | Alles wat gezegd wordt over de wensen m.b.t. digitalisering van informatie. |
| 5 Gewenste situatie | 5.2 Medimapp | 5.2.1. Functionaliteit | Alles wat gezegd wordt over de functionaliteiten die MediMapp in de gewenste situatie moet bevatten. |
|  |  | 5.2.2. Inhoud | Alles wat gezegd wordt over de specifieke inhoud van informatievoorziening wat in MediMapp moet zitten. |
|  |  | 5.2.3. Indruk | Alle eerste indrukken die worden beschreven door ouders en patiënten over MediMapp. |
|  |  | 5.2.4. Meerwaarde | Alles wat er wordt gezegd over de meerwaarde van MediMapp en het gevisualiseerde overzicht van het zorgtraject. |
|  |  | 5.2.5. Gebruik (suggestie) | Alle suggesties die worden verteld over het gebruik van MediMapp door de eindgebruikers. |

# 6 Communicatie voor kind

| Onderstaande factoren vallen onder dit domein:   - Beschrijving van de ervaringen op welk moment het onderwerp schisis wordt besproken met kind. - Beschrijving van manieren van communicatie met kind. |
| --- |

| **Domein** | **Subdomein** | **Code in MAXQDA** | **Omschrijving van de code** |
| --- | --- | --- | --- |
| 6 Communicatie voor kind | 6.1 Manieren (hoe?) | 6.1 Manieren (hoe?) | Alles wat er gezegd wordt over de manier waarop er met kinderen gecommuniceerd kan worden over hun aandoening. |
| 6 Communicatie voor kind | 6.2 Moment (leeftijd?) | 6.2 Moment (leeftijd?) | Alles wat er gezegd wordt over welke communicatie geschikt is voor welke leeftijd. |
| 6 Communicatie voor kind | 6.3 Inhoud | 6.3 Inhoud | Alles wat er gezegd wordt over de inhoud waarop er met het kind gecommuniceerd kan worden. |
| 6 Communicatie voor kind | 6.4 Lotgenoten | 6.4 Lotgenoten | Alles wat er gezegd wordt over lotgenoten betrekken in de communicatie met kinderen . |
